# The reflection of a blast wave by a very intense explosion

**DOI:** 10.1098/rspa.2021.0154

**Published:** 2021-06

**Authors:** Andrew W. Cook, Joseph D. Bauer, Gregory D. Spriggs

**Affiliations:** Lawrence Livermore National Laboratory, Livermore, CA, USA

**Keywords:** blast waves, numerical simulations, nuclear detonations, Taylor’s Equation, airdrops, surface detonations

## Abstract

We demonstrate that the geometric similarity of Taylor’s blast wave persists beyond reflection from an ideal surface. Upon impacting the surface, the spherical symmetry of the blast wave is lost but its cylindrical symmetry endures. As the flow acquires dependence on a second spatial dimension, an analytic solution of the Euler equations becomes elusive. However, the preservation of axisymmetry, geometric similarity and planar symmetry in the presence of a mirror-like surface causes all flow solutions to collapse when scaled by the height of burst (HOB) and the shock arrival time at the surface. The scaled blast volume for any yield, HOB and ambient air density follows a single universal trajectory for all scaled time, both before and after reflection.

## 1. Introduction

The Taylor [1,2], von Neumann & Richtmyer [3] and Sedov [4] solutions for a self-similar blast wave in the strong-shock limit have been used for eight decades to estimate the yields of nuclear tests and to explain the behaviour of supernovae [5], stellar wind bubbles [6] and other high-energy phenomena that produce strong shock waves [7,8]. During the atmospheric-testing era, weapon scientists employed Taylor’s equation to estimate the yields of 210 atmospheric tests by analysing high-speed camera films [9,10]. In most of these tests, the devices were detonated sufficiently high above the ground to ignore the shock interaction with the surface. In at least 14 of these events, however, the blast wave reached the ground too soon for Taylor’s equation to correctly apply to the data. Measured blast distances in these cases appear to exceed Taylor’s prediction due to reflected energy reinforcing the upward-moving shock. Consequently, their yields appear to be up to 40% higher than the yields determined by radiochemical techniques, light-curve analyses and other methods.

**Table 1.** Nomenclature.

M	denotes units of mass
L	denotes units of length
T	denotes units of time
[ = ]	‘has units of’
*Y*	total energy yield of device
*Y*_*a*_	portion of total yield that couples to air and forms blast wave
*Y*_*l*_	portion of total yield ‘lost’ to radiation, vaporization of bomb material, etc.
*ρ*_*a*_	ambient air density at burst location
*γ*	adiabatic index of air, aka ratio of specific heats
$S_{\gamma }$	Taylor’s similarity constant
*H*	height of burst (HOB)
*t*_*H*_	time when blast contacts surface
*V*_*H*_	blast volume at *t*_*H*_
*ρ*	density
Ω	geometric parameter
Θ	reflected-energy parameter
Ψ	reflection function
*e*	internal energy
*p*	pressure
*R*_*s*_	spherical blast radius according to Taylor’s solution
*t*	time since detonation
*V*	blast volume

**Figure 1.**
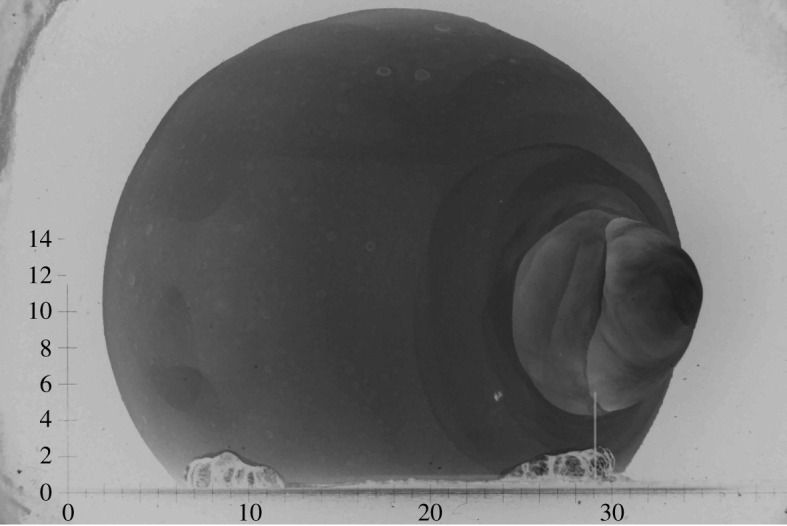
The Mohawk event at *t* = 0.010148 s. The bulbous jet on the right was caused by shielding placed alongside the device.

The Mohawk event, for example, exhibited a large discrepancy in the yield inferred by Edgerton, Germeshausen and Grier, Inc. (EG&G), when comparing the blast volume with the light output; i.e. the blast volume suggested a yield of approximately 360 kilotons whereas the light output indicated approximately 248 kilotons [11]. EG&G reasoned that spots on the fireball surface and the appearance of a bulbous jet, seen in figure 1, corrupted the light measurements; hence they rejected the light-curve data and reported the higher yield. EG&G, however, did not account for the reflection of the blast wave from the ground surface, which significantly increased the blast volume. The purpose of this paper is to lay out the additional reflection equations, which must be applied to film data in order to properly account for reflected blast energy. Throughout this paper, we use the term ‘blast’ to refer to the shock front, rather than the fireball surface, although the latter is typically easier to identify on the films.

## 2. Dimensional analysis

Taylor’s blast wave [1,2] for air with zero ambient energy and pressure is governed by only two parameters possessing physical dimensions; the energy of the point source

2.1 $$Y_{a}[=]\frac{ML^{\text{2}}}{T^{\text{2}}}$$

and the density of the air into which the energy is deposited

2.2 $$\rho_{a}[=]\frac{M}{L^{\text{3}}}.$$

Combining *Y*_*a*_ and *ρ*_*a*_ to eliminate M results in a single fixed relationship between L and T. In Taylor’s infinite atmosphere, where no intrinsic length or time scales exist, L and T remain locked together $\left(L^{\text{5}}\propto T^{\text{2}}\right)$, such that neither can abide independent of the other. The blast radius must therefore scale with its own time of propagation,

2.3 $$R_{s}=S_{\gamma }{\left(\frac{Y_{a}t^{2}}{\rho_{a}}\right)}^{1/5},$$

where the similarity constant $S_{\gamma }$ is solely a function of *γ*. The fixed relation between L and T means that the blast wave is completely specified either by its radius or time of propagation.

The presence of a boundary introduces a third dimensional parameter into the system, H[=]L, along with a time scale, $t_{H}[=]T$ associated with the new parameter. For a detonation at distance *H* above a perfectly reflecting (mirror-like) surface, the blast wave will reach the surface at time

2.4 $$t_{H}={\left[\frac{\rho_{a}}{Y_{a}}{\left(\frac{H}{S_{\gamma }}\right)}^{5}\right]}^{1/2}.$$

For *t* > *t*_*H*_, the fixed relation between L and T is broken, the spherical symmetry is lost and *R*_*s*_ ceases to parametrize the flow. However, the flow remains axisymmetric and the blast can still be fully characterized by its net volume, regardless of the complexity of the Mach stem and triple point emerging from the reflection. Since the ideal surface acts as a plane of symmetry, the flow behaves as if there were two identical blasts, separated a distance 2*H* apart; i.e. a blast and its mirror image [12].

Prior to reflection, the blast volume can be written in terms of (2.3)

2.5 $$V(t\leq t_{H})=\frac{4\pi }{3}R_{s}^{3}=\frac{4\pi }{3}S_{\gamma }^{3}{\left(\frac{Y_{a}t^{2}}{\rho_{a}}\right)}^{3/5}.$$

For *t*/*t*_*H*_ > 1, the blast wave gradually transforms from a sphere into a hemisphere, as energy is redirected back up from the surface. In the long-time limit, all of the blast energy is contained in a hemispherical volume. This long-term hemispherical volume, containing energy *Y*_*a*_ could also have arisen from an unbounded Taylor blast of energy 2*Y*_*a*_. Therefore, we can write the long-term solution as

2.6 $$V(t\gg t_{H})=\frac{2\pi }{3}S_{\gamma }^{3}{\left(\frac{2Y_{a}t^{2}}{\rho_{a}}\right)}^{3/5}.$$

Comparing (2.5) to (2.6), we see that a general solution takes the form

2.7 $$V(t)=\frac{4\pi }{3\Omega }S_{\gamma }^{3}{\left(\frac{\Theta Y_{a}t^{2}}{\rho_{a}}\right)}^{3/5},$$

where

2.8 $$\Omega ,\;\Theta =\left\{ \begin{array}{ll} 1 & \text{for}\;0\leq \frac{t}{t_{H}}\leq 1\\ 2 & \text{for}\;\frac{t}{t_{H}}\to \infty \end{array}\right..$$

Here we have introduced Ω to parametrize the geometric transformation of the blast from a sphere into a hemisphere, implicitly embedding all the intermediate complexities of the Mach stem and triple point associated with the shock-boundary interaction. The Θ parameter accounts for the energy reflected from the surface, including the time it takes for this energy to propagate back through the blast region and reach the perimeter. Since Ω and Θ obey the same limits, it is convenient to combine them into a single parameter

2.9 $$\frac{\Theta^{3/5}}{\Omega }\equiv \Psi^{-2/5}.$$

At *t* = *t*_*H*_, the blast volume is

2.10 $$V_{H}=\frac{4}{3}\pi H^{3}=\frac{4}{3}\pi S_{\gamma }^{3}{\left(\frac{Y_{a}t_{H}^{2}}{\rho_{a}}\right)}^{3/5}$$

and all reflections begin from the same non-dimensional initial conditions: *V*/*V*_*H*_ = 1 at *t*/*t*_*H*_ = 1. Subsequently, all flows, regardless of yield, height of burst (HOB) and/or ambient density, remain indistinguishable in non-dimensional variables. The collapse of flow solutions is a consequence of the fact that there is nothing in the initial conditions, boundary conditions or the equations themselves to break the symmetry or geometric similarity of the blast wave; hence, the temporal evolution of *V*/*V*_*H*_ does not depend on the shape of the blast wave. Dividing (2.7) by (2.10), we obtain a universal relationship between time and volume

2.11 $$\frac{V}{V_{H}}=\Psi^{-2/5}{\left(\frac{t}{t_{H}}\right)}^{6/5}.$$

The goal of this paper is to determine Ψ by performing computer simulations to directly calculate *V*.

## 3. Simulations

We have computed Ψ numerically using the Miranda code [13] to solve the governing Euler equations [14] for the transport of mass, momentum and energy. The Miranda code discretizes spatial derivatives with a tenth-order compact-finite-difference scheme [15] and temporally integrates the equations with a fourth-order Runge–Kutta method [16]. Eighth-order hyperviscosity [17] and hyperconductivity [18] are employed, along with eighth-order spectral-like dealiasing for shock capturing [19]. The blast volume is computed by tagging grid cells as the shock passes through them and then adding up the volumes of all the tagged cells.

The ideal-gas law assumed by Taylor and employed in our simulations is

3.1 p=(γ−1)ρe.

**Figure 2.**
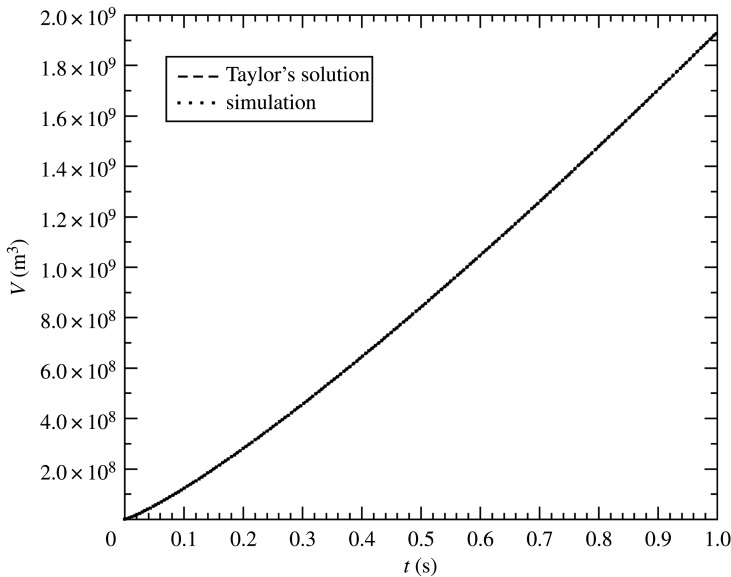
Blast volume versus time for a 100 kt detonation at 100 m HOB in a 1 kg m^−3^ density atmosphere.

In all of our simulations, we set *γ* = 1.2265, for which $S_{\gamma }=0.91952432$. We chose this value of gamma as a best fit to blast waves analysed in 43 atmospheric tests, including both tower shots and air bursts; i.e. we found this value to produce the best overall agreement between yields estimated from blast-wave measurements and radio-chemical methods. Before collecting results, we first verified that Miranda agrees with Taylor’s solution, given sufficient grid resolution. Here we present our grid-converged results obtained at a mesh spacing of *H*/800. Simulation versus theory for our benchmark case is displayed in figure 2.

## 4. Results

A useful means for comparing different cases is obtained by integrating (3.1) over all space

4.1 $$\int_{R^{3}}p\;\text{d}V=(\gamma -1)Y_{a}.$$

**Figure 3.**
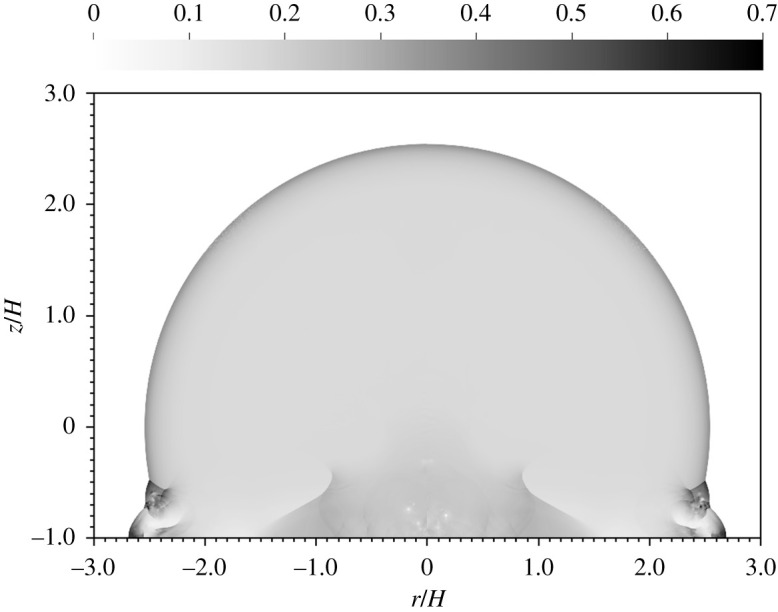
Non-dimensional pressure at *t*/*t*_*H*_ = 10 for a 100 kt detonation at 10 m HOB (left) versus 1 km HOB (right).

Since pressure becomes uniformly distributed at a sufficient distance behind the blast, the non-dimensional pressure, *pV*/*Y*_*a*_, provides a natural measure of correlation for our various simulations. Figure 3 displays the non-dimensional pressure from two different simulations, one at an HOB of 10 m and the other at an HOB of 1 km. In non-dimensional coordinates, the two blasts are seen to be identical, except for tiny differences arising from grid-seeded perturbations of the vorticity field. As the shock strikes the surface at an angle, it generates baroclinic vorticity, which in turn becomes Kelvin–Helmholtz unstable, leading to the formation of vortices with low-pressure cores, seen in the image near ground zero. The perturbations to the vorticity strip are generated by the numerical discretization but the subsequent evolution of the vortices is physical. The volume of the blast, however, is unaffected by these details.

**Figure 4.**
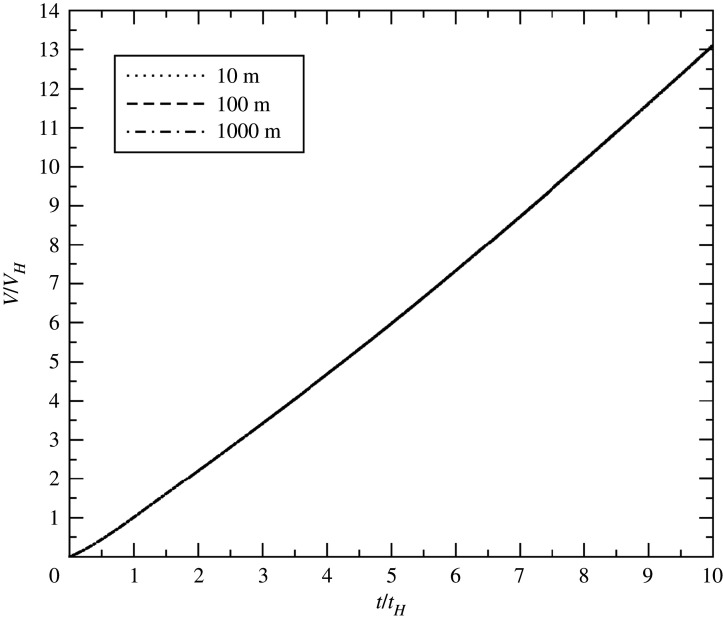
Non-dimensional volume versus non-dimensional time for a 100 kt detonation at 10, 100 and 1000 m HOB.

In figure 4, we plot scaled volume versus scaled time for a 100 kiloton detonation at three different HOBs spanning two orders of magnitude. The non-dimensionalized blast volumes are seen to collapse to a single curve. This is not too surprising, since we chose our reference time and volume to guarantee that all curves would pass through *V*/*V*_*H*_ = 1 at *t*/*t*_*H*_ = 1; i.e. changing the HOB can make no difference if time and volume are measured in HOB units.

**Figure 5.**
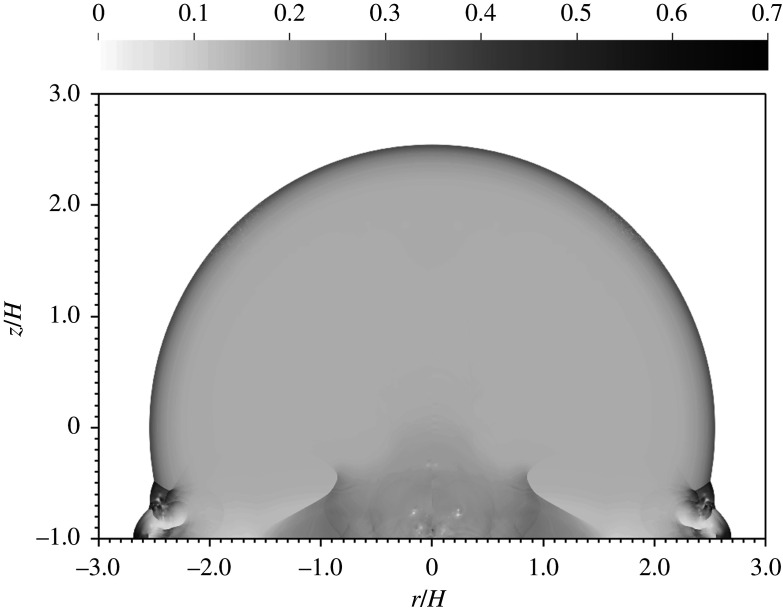
Non-dimensional pressure at *t*/*t*_*H*_ = 10 for a 10 kt detonation (left) versus a 1 Mt detonation (right) at 100 m HOB.

**Figure 6.**
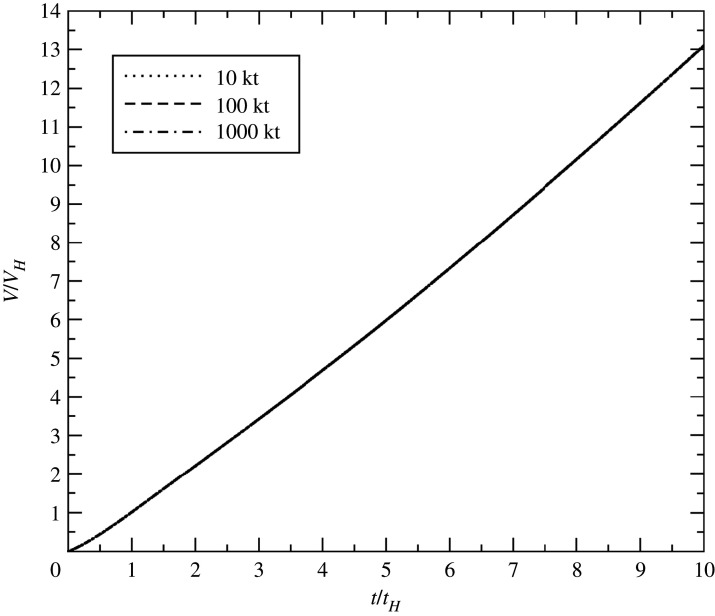
Non-dimensional volume versus non-dimensional time for 10 kt, 100 kt and 1 Mt detonations at 100 m HOB.

In figure 5, we compare different yields at the same HOB. Once again the blasts appear identical, even though the yields differ by two orders of magnitude. Changes in yield correspond to changes in blast velocity, but all velocities become equal in the chosen units, thus removing differences due to yield. Tiny variations are observed once more in the vortex cores near ground zero but they do not influence the blast volume. Scaled volume versus scaled time for three different yields is shown in figure 6. This single collapsed curve is identical to the one in figure 4. Although the yields and HOBs were varied in the simulations, they nevertheless all correspond to the same non-dimensional case.

**Figure 7.**
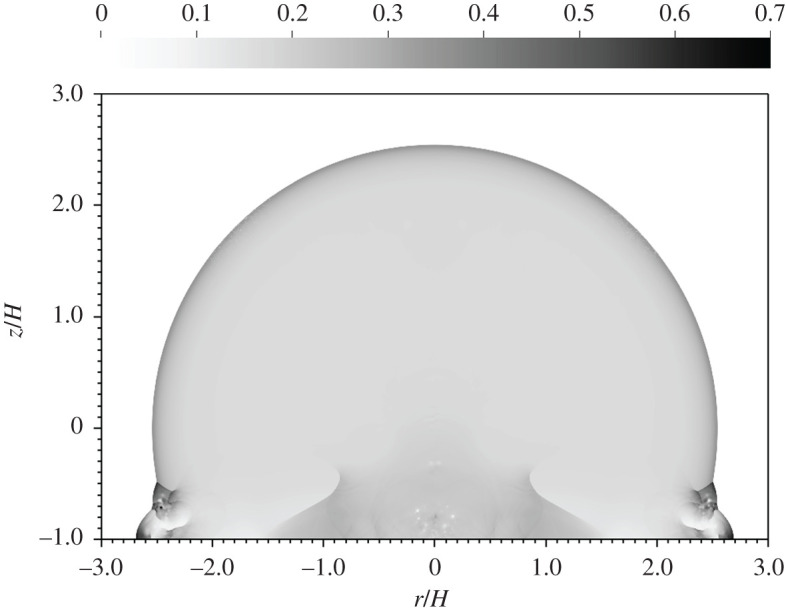
Non-dimensional pressure at *t*/*t*_*H*_ = 10 for a 100 kt detonation at 100 m HOB with ambient density 0.1 kg m^−3^ (left) versus 10 kg m^−3^ (right).

Finally, we varied the background density as an additional test of the theory. Figure 7 compares two simulations with atmospheric densities differing by two orders of magnitude, roughly one-tenth of an atmosphere to 10 atmospheres.

**Figure 8.**
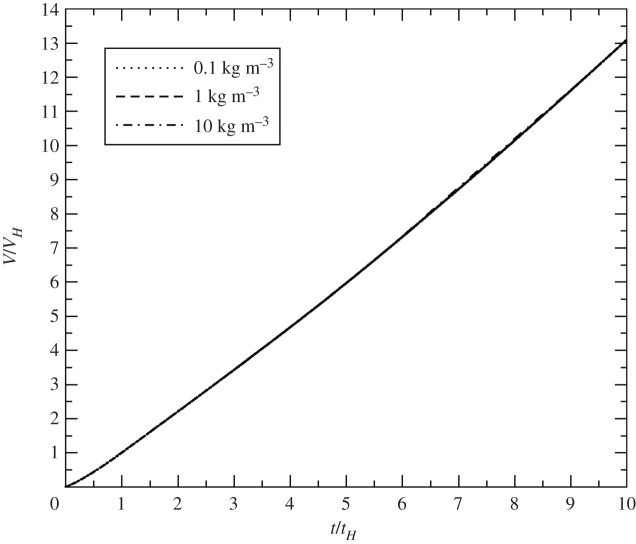
Non-dimensional volume versus non-dimensional time for a 100 kt detonation at 100 m HOB with ambient densities 0.1 kg m^−3^, 1 kg m^−3^ and 10 kg m^−3^.

The blasts appear identical, as they did with changes in yield and HOB. The volume versus time curves for three background densities are displayed in figure 8. The collapsed curve matches the previous cases.

We have thus far demonstrated that for any given yield, HOB and air density, the blast volume is a predictable function of time. In our simulations, the yield is a known input and the blast volume is calculated, however in applications of interest this process is reversed; i.e. the blast volume is measured and used to infer the yield. This becomes a straightforward procedure once the fixed relation between scaled volume and scaled time is established. Solving (2.11) for Ψ, we obtain the following result:

4.2 $$\begin{array}{rl} \Psi & ={\left(\frac{V_{H}}{V}\right)}^{5/2}{\left(\frac{t}{t_{H}}\right)}^{3}\\ & \approx 2-\exp(0.0269291\tau -0.374597\tau^{2}+0.108919\tau^{3}-0.0128575\tau^{4}), \end{array}$$

where

$$\tau \equiv \ln\left(\frac{t}{t_{H}}\right).$$

**Figure 9.**
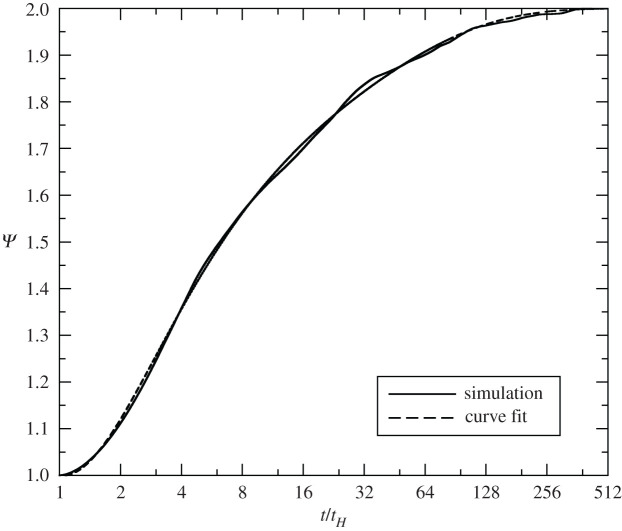
The reflection function (4.2) for all simulations.

We have plotted the reflection function (4.2) for all cases in figure 9. The undulations in the curve are due to the Mach stem and triple point working their way up the side of the blast wave. The curve fit has a correlation coefficient of 0.999345, an RMS relative error of 0.00481881 and a maximum relative error of 0.00756.

## 5. Conclusion

A universal reflection function exists for ideal blasts rebounding from perfect surfaces. We have computed this function from numerical simulations of Taylor blast waves with different yields, HOBs and ambient air densities. The reflection function relates non-dimensional blast volume to non-dimensional time, causing all cases to collapse onto a single curve. This curve serves as the basis for estimating the yield of tower shots and other nuclear events, wherein the cameras recorded the expanding blast wave after the shock reached the ground. Using the theory presented in this paper, it is possible to reconcile the EG&G data for Mohawk; i.e. if the reflection function is included in the blast wave analysis then the yield comes into agreement with the light-curve data and both methods give approximately 248 kilotons. This theory can be extended to include real-gas effects, radiation, humidity, gravity and energy-absorbing surfaces.

## Data accessibility

All relevant data are plotted in the figures and should be reproducible by other codes.
