# Supplementary material for: The reflection of a blast wave by a very intense explosion
Source: Proc Math Phys Eng Sci. 2021 Jun 2;477(2250):20210154. doi: 10.1098/rspa.2021.0154 (PMC8299555; doi:10.1098/rspa.2021.0154)
Supplement: Supplementary file 1 [file rspa20210154_review_history.pdf]

THE ROYAL SOCIETY
PUBLISHING

PROCEEDINGS A

The reflection of a blast wave by a very intense explosion

Andrew W. Cook, Joseph D. Bauer and Gregory D. Spriggs

Article citation details
Proc. R. Soc. A **477**: 20210154.
http://dx.doi.org/10.1098/rspa.2021.0154

Review timeline

Original submission:	19 February 2021
Revised submission:	30 April 2021
Final acceptance:	5 May 2021

Note: Reports are unedited and appear as submitted by the referee. The review history appears in chronological order.

Review History

RSPA-2021-0154.R0 (Original submission)

Review form: Referee 1

Is the manuscript an original and important contribution to its field?
Acceptable

Is the paper of sufficient general interest?
Acceptable

Is the overall quality of the paper suitable?
Excellent

Can the paper be shortened without overall detriment to the main message?
Yes

Do you think some of the material would be more appropriate as an electronic appendix?
No

Do you have any ethical concerns with this paper?
No

Recommendation?
Accept as is

Reports © 2021 The Reviewers; Decision Letters © 2021 The Reviewers and Editors; Responses © 2021 The Reviewers, Editors and Authors. Published by the Royal Society under the terms of the Creative Commons Attribution License http://creativecommons.org/licenses/by/4.0/, which permits unrestricted use, provided the original author and source are credited

Comments to the Author(s)
The manuscript does a good job of describing the problem and its solution. It does not seem surprising that the reflection from an ideal, frictionless surface would yield a nearly universal solution, since the reflected wave exactly corresponds to an identical, second blast wave. Only if baroclinic vorticity or other nonlinear effects are large would the reflection not be nearly self-similar.

If the lower atmosphere was strongly stratified, either naturally or by the popcorn effect from the blast radiation, the results might be a little different.

Review form: Referee 2

Is the manuscript an original and important contribution to its field?
Excellent

Is the paper of sufficient general interest?
Good

Is the overall quality of the paper suitable?
Excellent

Can the paper be shortened without overall detriment to the main message?
Yes

Do you have any ethical concerns with this paper?
No

Recommendation?
Accept with minor revision (please list in comments)

Comments to the Author(s)
p. 1: The authors state that in 14 events the blast wave interacted with the ground to alter the rate of expansion of the fireball. I believe the actual number from the US testing program is much higher (~39 events), based on the scaled height of burst of these events. I suggest changing the wording to "... in at least 14 of these events ..."

Reference 11, which (I believe) is critical to laying a concrete example of the practical importance of this work, is not generally available. Could it be provided as an electronic appendix?

p. 4: The author's state that "the blast gradually transforms from a sphere into a hemisphere ...". I was thinking about the Grable event, in which the reflected shock flattens the bottom portion of the fireball and it does not form into a hemisphere. But then I thought that perhaps the author's are thinking about the shock front itself (as opposed to the fireball front) as the "blast" and then it is that portion that forms into a hemisphere. If that is the case, perhaps a simple clarifying statement that by "blast" they mean the shock front. (?)

Equation (2.6): It is certainly obvious that this equation is applicable in the case of a surface burst, but less obvious to me that this equation should also be applicable in the general case of a detonation at any arbitrary height of burst. Perhaps the key is in the phrase "long-term limit", as stated. It could certainly be stated as an ansatz and the rest follows as presented.

Decision letter (RSPA-2021-0154.R0)

28-Apr-2021

Dear Dr Cook,

On behalf of the Editor, I am pleased to inform you that your Manuscript RSPA-2021-0154 entitled "The Reflection of a Blast Wave by a Very Intense Explosion I. Scaling and Similarity" has been accepted for publication subject to minor revisions in Proceedings A. Please find the referees' comments below.

The reviewer(s) have recommended publication, but also suggest some minor revisions to your manuscript. Therefore, I invite you to respond to the reviewer(s)' comments and revise your manuscript. Please note that we have a strict upper limit of 28 pages for each paper. Please endeavour to incorporate any revisions while keeping the paper within journal limits. Please note that page charges are made on all papers longer than 20 pages. If you cannot pay these charges you must reduce your paper to 20 pages before submitting your revision. Your paper has been ESTIMATED to be 11 pages. We cannot proceed with typesetting your paper without your agreement to meet page charges in full should the paper exceed 20 pages when typeset. If you have any questions, please do get in touch.

It is a condition of publication that you submit the revised version of your manuscript within 7 days. If you do not think you will be able to meet this date please let me know in advance of the due date.

To revise your manuscript, log into https://mc.manuscriptcentral.com/prsa and enter your Author Centre, where you will find your manuscript title listed under "Manuscripts with Decisions." Under "Actions," click on "Create a Revision." Your manuscript number has been appended to denote a revision.

You will be unable to make your revisions on the originally submitted version of the manuscript. Instead, revise your manuscript and upload a new version through your Author Centre.

When submitting your revised manuscript, you will be able to respond to the comments made by the referee(s) and upload a file "Response to Referees" in Step 1: "View and Respond to Decision Letter". You can use this to document any changes you make to the original manuscript. In order to expedite the processing of the revised manuscript, please be as specific as possible in your response to the referee(s).

IMPORTANT: Your original files are available to you when you upload your revised manuscript. Please delete any redundant files before completing the submission process.

When uploading your revised files, please make sure that you include the following as we cannot proceed without these:

1) A text file of the manuscript (doc, txt, rtf or tex), including the references, tables (including captions) and figure captions. Please remove any tracked changes from the text before submission. PDF files are not an accepted format for the "Main Document".

2) A separate electronic file of each figure (tif, eps or print-quality pdf preferred). The format should be produced directly from original creation package, or original software format.

3) Electronic Supplementary Material (ESM): all supplementary materials accompanying an accepted article will be treated as in their final form. Note that the Royal Society will not edit or typeset supplementary material and it will be hosted as provided. Please ensure that the

supplementary material includes the paper details where possible (authors, article title, journal name). Supplementary files will be published alongside the paper on the journal website and posted on the online figshare repository (https://figshare.com). The heading and legend provided for each supplementary file during the submission process will be used to create the figshare page, so please ensure these are accurate and informative so that your files can be found in searches. Files on figshare will be made available approximately one week before the accompanying article so that the supplementary material can be attributed a unique DOI.
Alternatively you may upload a zip folder containing all source files for your manuscript as described above with a PDF as your "Main Document". This should be the full paper as it appears when compiled from the individual files supplied in the zip folder.

Article Funder

Please ensure you fill in the Article Funder question on page 2 to ensure the correct data is collected for FundRef (http://www.crossref.org/fundref/).

Media summary

Please ensure you include a short non-technical summary (up to 100 words) of the key findings/importance of your paper. This will be used for to promote your work and marketing purposes (e.g. press releases). The summary should be prepared using the following guidelines:

*Write simple English: this is intended for the general public. Please explain any essential technical terms in a short and simple manner.
*Describe (a) the study (b) its key findings and (c) its implications.
*State why this work is newsworthy, be concise and do not overstate (true 'breakthroughs' are a rarity).
*Ensure that you include valid contact details for the lead author (institutional address, email address, telephone number).

Cover images

We welcome submissions of images for possible use on the cover of Proceedings A. Images should be square in dimension and please ensure that you obtain all relevant copyright permissions before submitting the image to us. If you would like to submit an image for consideration please send your image to proceedingsa@royalsociety.org

Open Access

You are invited to opt for open access, our author pays publishing model. Payment of open access fees will enable your article to be made freely available via the Royal Society website as soon as it is ready for publication. For more information about open access please visit https://royalsociety.org/journals/authors/open-access/. The open access fee for this journal is £1700/$2380/€2040 per article. VAT will be charged where applicable. Please note that if the corresponding author is at an institution that is part of a Read and Publishing deal you are required to select this option. See https://royalsociety.org/journals/librarians/purchasing/read-and-publish/read-publish-agreements/ for further details.

Once again, thank you for submitting your manuscript to Proceedings A and I look forward to receiving your revision. If you have any questions at all, please do not hesitate to get in touch.

Best wishes
Raminder Shergill
proceedingsa@royalsociety.org
Proceedings A

Reviewer(s)' Comments to Author:

Referee: 1

Comments to the Author(s)
The manuscript does a good job of describing the problem and its solution. It does not seem surprising that the reflection from an ideal, frictionless surface would yield a nearly universal solution, since the reflected wave exactly corresponds to an identical, second blast wave. Only if baroclinic vorticity or other nonlinear effects are large would the reflection not be nearly self-similar.

If the lower atmosphere was strongly stratified, either naturally or by the popcorn effect from the blast radiation, the results might be a little different.

Referee: 2

Comments to the Author(s)
p. 1: The authors state that in 14 events the blast wave interacted with the ground to alter the rate of expansion of the fireball. I believe the actual number from the US testing program is much higher (~39 events), based on the scaled height of burst of these events. I suggest changing the wording to "... in at least 14 of these events ..."

Reference 11, which (I believe) is critical to laying a concrete example of the practical importance of this work, is not generally available. Could it be provided as an electronic appendix?

p. 4: The author's state that "the blast gradually transforms from a sphere into a hemisphere ...". I was thinking about the Grable event, in which the reflected shock flattens the bottom portion of the fireball and it does not form into a hemisphere. But then I thought that perhaps the author's are thinking about the shock front itself (as opposed to the fireball front) as the "blast" and then it is that portion that forms into a hemisphere. If that is the case, perhaps a simple clarifying statement that by "blast" they mean the shock front. (?)

Equation (2.6): It is certainly obvious that this equation is applicable in the case of a surface burst, but less obvious to me that this equation should also be applicable in the general case of a detonation at any arbitrary height of burst. Perhaps the key is in the phrase "long-term limit", as stated. It could certainly be stated as an ansatz and the rest follows as presented.

Decision letter (RSPA-2021-0154.R1)

05-May-2021

Dear Dr Cook

I am pleased to inform you that your manuscript entitled "The Reflection of a Blast Wave by a Very Intense Explosion" has been accepted in its final form for publication in Proceedings A.

Our Production Office will be in contact with you in due course. You can expect to receive a proof of your article soon. Please contact the office to let us know if you are likely to be away from e-mail in the near future. If you do not notify us and comments are not received within 5 days of sending the proof, we may publish the paper as it stands.

As a reminder, you have provided the following 'Data accessibility statement' (if applicable). Please remember to make any data sets live prior to publication, and update any links as needed when you receive a proof to check. It is good practice to also add data sets to your reference list. Statement (if applicable): All relevant data are plotted in the figures and should be reproducible by other codes.

Open access
You are invited to opt for open access, our author pays publishing model. Payment of open access fees will enable your article to be made freely available via the Royal Society website as soon as it is ready for publication. For more information about open access please visit https://royalsociety.org/journals/authors/which-journal/open-access/. The open access fee for this journal is £1700/$2380/€2040 per article. VAT will be charged where applicable.

Note that if you have opted for open access then payment will be required before the article is published – payment instructions will follow shortly.

If you wish to opt for open access then please inform the editorial office (proceedingsa@royalsociety.org) as soon as possible.

Your article has been estimated as being 11 pages long. Our Production Office will inform you of the exact length at the proof stage.

Proceedings A levies charges for articles which exceed 20 printed pages. (based upon approximately 540 words or 2 figures per page). Articles exceeding this limit will incur page charges of £150 per page or part page, plus VAT (where applicable).

Under the terms of our licence to publish you may post the author generated postprint (ie. your accepted version not the final typeset version) of your manuscript at any time and this can be made freely available. Postprints can be deposited on a personal or institutional website, or a recognised server/repository. Please note however, that the reporting of postprints is subject to a media embargo, and that the status the manuscript should be made clear. Upon publication of the definitive version on the publisher's site, full details and a link should be added.

You can cite the article in advance of publication using its DOI. The DOI will take the form: 10.1098/rspa.XXXX.YYYY, where XXXX and YYYY are the last 8 digits of your manuscript number (eg. if your manuscript number is RSPA-2017-1234 the DOI would be 10.1098/rspa.2017.1234).

For tips on promoting your accepted paper see our blog post: https://royalsociety.org/blog/2020/07/promoting-your-latest-paper-and-tracking-your-results/

On behalf of the Editor of Proceedings A, we look forward to your continued contributions to the Journal.

Sincerely,
Raminder Shergill
proceedingsa@royalsociety.org
